# Acute dizziness and mental alteration associated with Moderna COVID-19 vaccine: a case report

**DOI:** 10.1186/s12883-022-02834-8

**Published:** 2022-08-26

**Authors:** Rizaldy Taslim Pinzon, Fillia Kristyawati Haryono, Nikolaus Erik Darmawan, Mia Amelia Mutiara Salikim, Vanessa Veronica

**Affiliations:** 1 Duta Wacana Christian University School of Medicine, Yogyakarta, Indonesia; 2 Bethesda Lempuyangwangi Hospital, Yogyakarta, Indonesia; 3 Bethesda Hospital, Yogyakarta, Indonesia

**Keywords:** Moderna, Vaccine, Acute dizziness, Mental alteration, Encephalopathy

## Abstract

**Background:**

Due to a rising number of COVID-19 cases, the Indonesian government implemented public health programs to lower the rate. Since January 2021, one of the government’s primary policies has been the COVID-19 immunization program. Recently, the Moderna messenger ribonucleic acid (mRNA) vaccine is one of the COVID-19 vaccines used in Indonesia. Based on some research, Moderna has possible side effects throughout the body, including neurological symptoms.

**Case presentation:**

We describe a 39-year-old female with uncontrolled hypertension who showed behavioral change, communication difficulty, social withdrawal, and a confused state within 7 days from getting her first dose of the Moderna vaccine. The patient had a history of febrile convulsion in childhood. An increase of neutrophil-to-lymphocyte ratio (16.9) and C-reactive protein level (31.75 mg/L) indicates ongoing inflammation. Head CT scan shows no abnormalities. She received ceftriaxone, citicoline, and methylprednisolone. The patient was discharged on the seventh day and completely recovered 1 week later. This study is the first case report of encephalopathy following the administration of the Moderna COVID-19 vaccine reported in Indonesia up to our knowledge.

**Conclusion:**

Encephalopathy related to the Moderna COVID-19 vaccine should be acknowledged as an adverse effect of the Moderna COVID-19 vaccine.

## Background

COVID-19 instances are growing in Indonesia, making it one of the worldwide epicenters in July 2021. Since January 2021, the government has been promoting the COVID-19 immunization campaign. Moderna is a COVID-19 vaccination used in Indonesia. The Moderna COVID-19 vaccine was 94.1% effective in preventing COVID-19 cases in the third phase clinical study [1]. Vaccine adverse effects range from moderate to severe. Brain-fogging symptoms (9.95%), vertigo-like symptoms (3.47%), coordination disturbance (0.93%), paralysis (0.69%), and seizures (0.69%) were reported by 1116 individuals (0.23%) [2].

Encephalopathy is a brain dysfunctional state associated with neurological and psychiatric disorders. Clinical manifestations of encephalopathy are cognitive dysfunction, behavioral change, disorientation, tremor, paralysis, and seizure [3]. Research on the side effects of the Moderna COVID-19 vaccine on the nervous system is still limited, including encephalopathy cases. We report a case of encephalopathy in a patient following Moderna COVID-19 vaccine administration.

## Case presentation

A 39-year-old female was hospitalized at Bethesda Lempuyangwangi Hospital Yogyakarta Indonesia, with the chief complaints of confused state, dizziness, nausea, vomiting, and epigastric pain. Symptoms began 1 day before hospitalization. Behavioral changes, communication difficulties, social disengagement, and confusion were reported by the patient’s family 1 day before admission. The patient has uncontrolled hypertension and a history of uncomplicated febrile seizures at age one. She has never taken any antiepileptic medication and has never suffered recurrent seizures. The family denied a history of mental illness, smoking, alcohol, and illegal drug use. The patient had 2 doses of Sinovac vaccine on January 2021 and February 2021 and had 1 dose of Moderna COVID-19 vaccine on August 20th, 2021. On the following day, the patient experienced tenderness and swelling on the Moderna COVID-19 vaccine administration site and subsided after the paracetamol consumption.

**Fig. 1:**
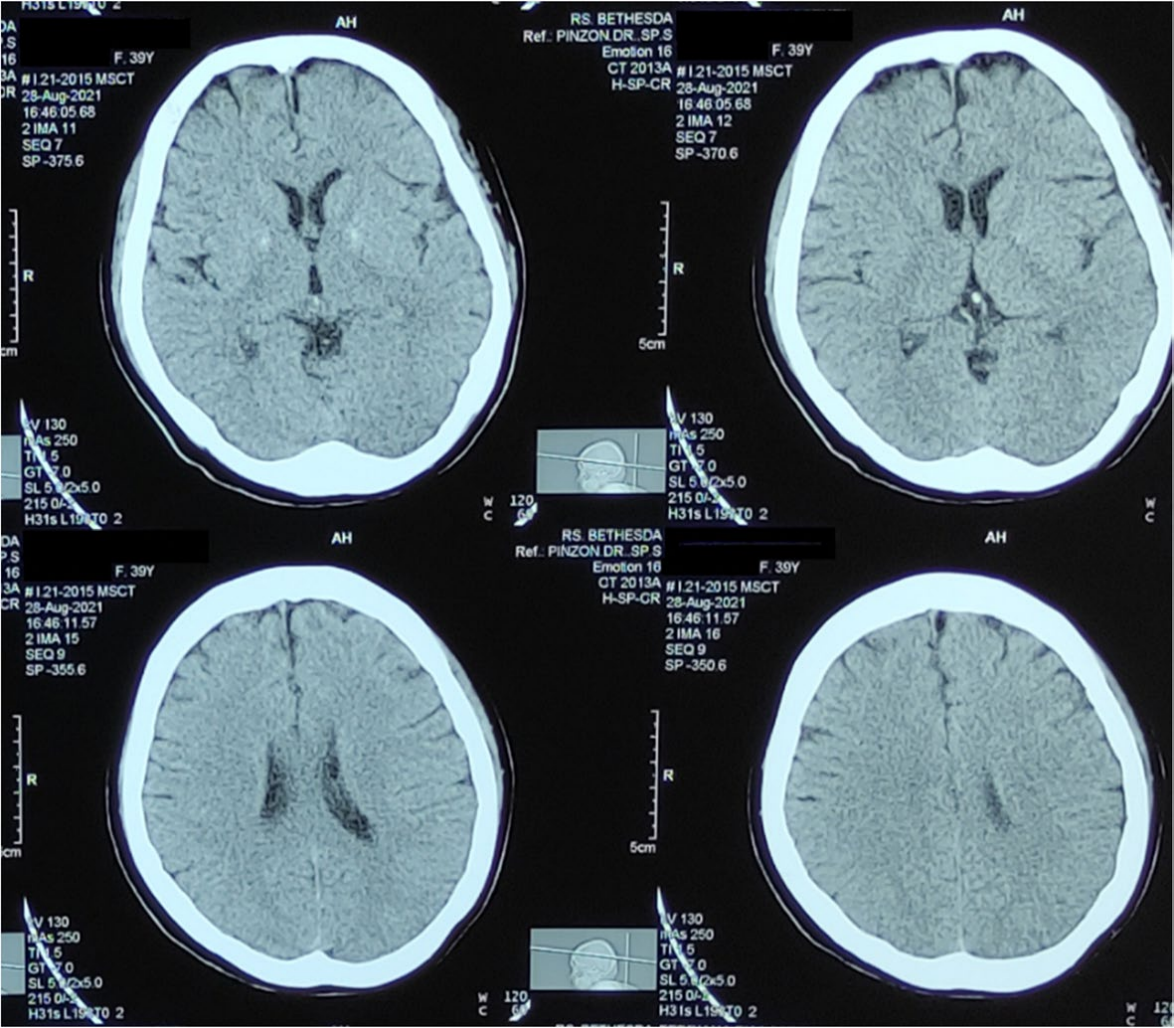
Head CT-scan examination shows no abnormalities

An emergency room evaluation found 160/85 mmHg blood pressure, 22 breaths per minute respiratory rate, 100 beats per minute pulse rate, no fever, and 97% oxygen saturation on room air. Memory loss, difficulty pronouncing words, impaired numeracy and linguistic skills, time and place disorientation, and attention deficiency were all noted in the ER. Examiners did not find any cranial nerve palsy, motor and sensory deficit, pathological reflexes, and meningeal signs. The neutrophil-to-lymphocyte ratio (16.9) and C-reactive protein (31.75) increased, indicating an ongoing inflammation (Table 1). A chest x-ray and urinalysis revealed no abnormalities. The COVID-19 polymerase chain reaction (PCR) test came out negative. The computed tomography (CT) scan of the head revealed no anomalies (Fig. 1). Due to our hospital’s facility limitations, we did not undertake brain MRI, electroencephalography, or cerebrospinal study. Moderna COVID-19 vaccine-associated encephalopathy was diagnosed. The patient received 1 g ceftriaxone twice a day, 40 mg pantoprazole once daily, 4 mg ondansetron three times daily, 5 mg flunarizine twice daily, and 12 mg betahistine three times daily.

**Table 1:** Laboratory Findings

Hemoglobin level	11.1 g/dL
Platelet count	278 × 10^3^/μL
Leukocyte count	9.31 × 10^9^/L
Neutrophil	90.6%
Lymphocyte	5.4%
neutrophil-to-lymphocyte ratio	16.9
Random blood glucose	128 mg/dL
C-reactive protein	31.75 mg/dL
Sodium level	134 mEq/L
Potassium level	3.7 mEq/L
Total cholesterol level	196 mg/dL
Triglyceride level	136 mg/dL

On the second day of hospitalization, epigastric and left hypochondriac pain was found, but no neurological abnormality. As a therapy, 62.5 mg of methylprednisolone injection twice daily was added. She began to recognize and interact with her surroundings on her third day in the hospital. When the patient was compliant, an MMSE was performed. The MMSE score on the third and fourth day was 18, indicating slight cognitive impairment. The sixth day of hospitalization showed a significant decrease in C-reactive protein to 10.21 mg/L. The patient was discharged on the seventh day and there were no adverse events observed. One week following hospital discharge, the patient was recovered completely. We re-administered the MMSE 10 months later, and her score of 30 shows that she does not have cognitive impairment.

## Discussion and conclusion

This study is the first case report of encephalopathy following the administration of the Moderna COVID-19 vaccine reported in Indonesia up to our knowledge. Several studies have already reported the side effects of the Moderna COVID-19 vaccine. The pathophysiology and the underlying mechanism of encephalopathy after the Moderna COVID-19 vaccine were still unknown [4].

Interestingly, our patient’s symptoms parallel with previous studies [5, 6], including behavioral changes, severe disorientation, and cognitive deficits. These symptoms emerged 2–7 days following the Moderna COVID-19 vaccine. All of the patients presented with normal findings on the Head CT-scan and brain MRI but increased the C-reactive protein level indicating an ongoing inflammation. The patients’ RT-PCR SARS-CoV-2 tests likewise came back negative, indicating no COVID-19 acute infection. Similar conditions and symptoms also have been reported in patients after receiving the Moderna COVID-19 vaccine in another study, which outlined three patients with encephalopathy after getting the the vaccine [7].

Prior research reported neurological adverse effects following coronavirus 2019 vaccination per one million vaccination doses administered within 42 days in the United States of America. The most-reported neurological side effects were headache, fatigue, dizziness, tinnitus, and sleep disruption. Several serious neurological problems were also reported, such as encephalopathy, visual loss, syncope, seizure, Bell’s palsy, and ischemic stroke. The incidence of encephalopathy was 10.36 per one million doses of vaccination. Interestingly, the incidence reported by the research varied depending on the vaccination. The incidence of encephalopathy induced by Pfizer, Moderna, and Janssen per 1,000,000 vaccination doses was 7.80; 10.04; 49.89. These data highlight the potential neurological side effects of the COVID-19 vaccination, specifically the Moderna COVID-19 vaccine [8].

The inhibition of brain-derived neurotropic factor (BDNF) might play a role in developing neuropsychiatry symptoms in COVID-19 cases. Spike (S) Glycoprotein is a SARS-CoV-2 cell-surface protein that mediates the virus binding on Angiotensin-Converting Enzyme-2 (ACE-2) Receptor. This binding may decrease the activity of brain-derived neurotrophic factor (BDNF)*.* Theoretically, the decrease of BDNF will subsequently reduce the anti-inflammatory action of BDNF on neurons and attenuate microglial activation. The increase of inflammatory signals may increase the likelihood of seizures and other related neurological complications. The decrease of BDNF is also related to cognitive impairment. Another possible explanation is COVID-19 patients may experience an increase in cytokines such as *interleukin-6* (IL-6) and *tumor necrosis factor-*α (TNF-α). Those cytokines enable to pass through the blood-brain barrier and activate microglia. This activation produces interleukin-1 (IL-1), which receptor is highly concentrated in the hippocampus, hence impairing memory and attention [9].

Moderna COVID-19 vaccine uses S-2P antigen to stabilize spike glycoprotein. The S1 and S2 subunit will stay associated until binding to the ACE-2 receptor via the Receptor-Binding Domain (RBD) [10]. In theory, spike protein synthesis might initiate the same inflammatory cascade as COVID-19 [6]. Although our patient had a history of simple febrile seizures, it is well-known that these have a favourable prognosis and do not result in neurologic or cognitive impairment over the long run [11].

Two days after the steroid administration, our patient had a dramatic improvement in cognitive function, shown by the significant increase of MMSE score. This situation shares a similarity with the previous study*,* in which the patient also had a dramatic improvement after the administration of methylprednisolone [5]. It is too early to conclude the efficacy and effectiveness of steroids in this typical case and need further studies to investigate the association.

This case report has some limitations since the patient did not undergo brain MRI examination, electroencephalography, and cerebrospinal fluid analysis due to the facility limitation on our hospital. Thus, any other etiologies cannot be entirely ruled out. We suggest a thorough examination for further studies to investigate causality between the Moderna COVID-19 vaccine and acute encephalopathy.

In conclusion, encephalopathy related to the Moderna COVID-19 vaccine should be acknowledged as an adverse effect of the Moderna COVID-19 vaccine. This case report is likely to serve as a model for future research to establish links between the Moderna COVID-19 vaccination and encephalopathy.

## Data Availability

The data generated for this study are accessible upon request to the first author, Rizaldy Taslim Pinzon (drpinzon17@gmail.com).
